# Genetic commonalities between rare subtypes of ALS and CMT: insights into molecular mechanisms of neurodegeneration

**DOI:** 10.1007/s00726-026-03500-w

**Published:** 2026-02-01

**Authors:** Abdilatif Aynaashe, Petri Kursula

**Affiliations:** 1 Department of Biomedicine, University of Bergen, Bergen, Norway; 2 Faculty of Biochemistry and Molecular Medicine, University of Oulu, Oulu, Finland; 3 LINXS Institute of Advanced Neutron and X-Ray Science, Lund, Sweden

**Keywords:** CMT, ALS, RNA transport and metabolism, Stress response dysfunction, Axonal transport dysfunction, Mitochondrial dysfunction, Protein aggregation, Molecular mechanism, Neurodegeneration

## Abstract

Amyotrophic lateral sclerosis (ALS) and Charcot-Marie-Tooth disease (CMT) are two distinct neurodegenerative disorders. While ALS is characterised by rapidly progressive motor neuron degeneration, leading to severe complications and death, CMT as a peripheral neuropathy is less severe, and patients have a longer life span, although with a compromised quality of life. Despite their clinical differences, current knowledge suggests that familial ALS (fALS) and CMT may share common genetic and molecular mechanisms. We aimed to identify shared genes mutations and molecular pathways between fALS and CMT through a literature and database search. Thirteen genes were identified, involved in distinct cellular processes: axonal transport (*DYNC1H1*,* KIF5A*,* SPG11*, *DCTN1*), protein homeostasis (*NEFH*,* VCP*,* SOD1*), RNA metabolism (*GARS*,* SETX*), cellular stress response (*HSPB1*,* FIG4*), and mitochondrial function (*MFN2*, *CHCHD10*). While these linkages to the two diseases are rare for each gene, understanding possible mechanistic commonalities at the molecular level can initiate new research directions, help in identifying additional common genes between neurodegenerative disorders, and improve diagnostics.

## Background

Neurodegenerative diseases encompass a broad range of disorders characterised by progressive neuronal dysfunction and degeneration. Amyotrophic lateral sclerosis (ALS) and Charcot-Marie-Tooth disease (CMT) represent two distinct conditions that affect motor and peripheral neurons, respectively. While classified as separate disorders, emerging evidence suggests the presence of common genetic and molecular mechanisms in familial ALS (fALS) and CMT, challenging conventional disease classification models (Martin et al. 2020).

Both ALS and CMT were first described by the French neurologist Jean-Martin Charcot in the mid-to-late 19th century. Attempting to correlate clinical findings in neurological patients with anatomical discoveries, he classified multiple key neurological disorders, including ALS, CMT, and multiple sclerosis (MS). His work predated the modern understanding of inheritance, molecular biology, and genetics; only over a century later, the first genotype-phenotype correlations were made for CMT (*PMP22*) in 1991 (Li et al. 2013) and for ALS (*SOD1*) in 1993 (Siddique and Ajroud-Driss 2011).

ALS was first documented by Charcot in 1869 and became widely known in 1939 following the diagnosis of the famous baseball player Lou Gehrig. Therefore, ALS is commonly referred to as *Lou Gehrig’s disease*. It gained further attention with the Ice Bucket Challenge in the summer of 2014, a global campaign that raised awareness and funds for ALS research. Another widely recognised figure associated with the condition is the renowned British physicist Stephen Hawking, who was diagnosed in 1963 and remarkably lived with ALS for more than five decades before passing away in 2015 at the age of 76 (Dobson 2002).

CMT was independently described by Charcot and his pupil Jean Marie in France and Howard Tooth in the UK in 1886, with Tooth correctly localising the pathology to peripheral nerves; Charcot and Marie thought the lesion was in the spinal cord (Kazamel and Boes 2015). There have been challenges in accurately classifying neuropathies, and new proposals continue to emerge on how to best categorise CMT subtypes (Kazamel and Boes 2015; Mathis et al. 2015). The fact that mutations have been identified in > 100 genes causing different CMT subtypes highlights the heterogeneity of this condition at the molecular level.

We set out to investigate whether specific genes and molecular mechanisms could be shared between fALS and CMT more frequently than previously recognised, which could further shed light on common pathogenic pathways of neurodegeneration. In the following sections, the characteristics of ALS and CMT are presented based on a review of the literature. We shall briefly present both CMT and ALS from a clinical perspective. We will then further delve into the molecular world, aiming to understand the molecular basis of disease in both cases at the protein level, based on the literature and genetics databases. Possible common molecular and cellular denominators for fALS and CMT are discussed.

## Main text

### Amyotrophic lateral sclerosis

ALS is a fatal, progressive neurodegenerative disorder that affects upper and lower motor neurons in the motor cortex, brainstem, and spinal cord, leading to muscle weakness, paralysis, and respiratory failure (Masrori and Van Damme 2020). ALS can be classified based on clinical presentation, genetic causes, and other associated factors (Brooks et al. 2000; Grad et al. 2017). The two primary categories are sporadic ALS (sALS) and fALS. Most cases are sporadic (90%), occurring without a known family history. fALS, accounting for 10% of cases, is primarily autosomal dominant but can have recessive or X-linked patterns, depending on the gene involved (Grad et al. 2017).

ALS presents with heterogeneous clinical manifestations that can be categorised into subtypes, with two-thirds showing classical ALS (Masrori and Van Damme 2020). ALS-Plus syndromes refer to ALS with additional features, such as frontotemporal dementia (FTD) (common with *C9orf72* mutations), sensory loss, autonomic dysfunction, or parkinsonism (McCluskey et al. 2009).

Globally, the incidence and prevalence of ALS are increasing. The incidence ranges from 0.6 to 3.8 per 100 000 person-years, with higher rates in Europe (2.1–3.8 / 100 000). The prevalence varies between 4.1 and 8.4 / 100 000 person-years (Longinetti and Fang 2019). In Norway, the incidence has reached 3 per 100 000, with a prevalence of 6.9–7.6 per 100 000 from 2009 to 2015 (Olsen et al. 2022). This increase is likely due to better diagnosis and disease awareness (Longinetti and Fang 2019).

Over 50 genes have been associated with ALS (Mejzini et al. 2019), with *C9orf72*,* SOD1*,* TARDBP (TDP-43)*, and *FUS* being most commonly affected (Mejzini et al. 2019; Gall et al. 2020). The major ALS genes and their protein products are introduced in Table 1. ALS involves oxidative stress, excitotoxicity, mitochondrial dysfunction, impaired proteostasis, and neuroinflammation. Environmental risk factors may include contact sports, military service, smoking, heavy metals, pesticides and neurotoxins, and electromagnetic fields (Ingre et al. 2015).

**Table 1:** Overview of key genes in ALS

Gene name	Function	Pathology	Associated diseases	OMIM entry
*C9orf72 (most common) (*Farg et al. 2014)	Endosomal trafficking, Autophagy	Abnormal repeat expansion causes toxic protein aggregation(Grad et al. 2017)	ALS1, FTD	*614,260
*SOD1 (the first gene associated with ALS) (*Bunton-Stasyshyn et al. 2015)	Converts superoxide radicals to oxygen and hydrogen peroxide	Misfolded proteins lead to oxidative stress and neurotoxicity	ALS, STAHP (progressive spastic tetraplegia and axial hypotonia)	*147,450
*TDP-43 (*Jo et al. 2020)	RNA processing, Regulation of gene expression	Dysfunctional splicing leads to protein aggregation and cell toxicity	ALS10, with or without FTD, FTLD	*605,078
*FUS (*Deng et al. 2014)	RNA processing	Disrupted RNA-binding proteins lead to neuronal dysfunction	ALS6, with or without FTD, Essential tremor	*137,070

A combination of upper motor neuron (UMN) and lower motor neuron (LMN) symptoms is observed in most patients. Early signs include muscle atrophy and weakness, spasticity in the arms and legs, and hyperreflexia (Masrori and Van Damme 2020). Other symptoms include slurred speech, difficulty swallowing, respiratory problems, and emotional lability (Grad et al. 2017; Masrori and Van Damme 2020). The age of onset is 40–65 years (Masrori and Van Damme 2020). ALS is rapidly progressive, with a median survival of 2–5 years post-diagnosis (Masrori and Van Damme 2020). Patients may exhibit cognitive impairment, either mild or full FTD (McCluskey et al. 2009).

ALS is diagnosed based on clinical findings using the El Escorial criteria, which assess UMN and LMN signs and the absence of other explanatory diseases (Brooks et al. 2000). Electromyography (EMG) confirms LMN involvement, and genetic testing may identify known ALS mutations, particularly in fALS. Magnetic resonance (MR) imaging excludes structural lesions affecting the motor nervous system (Masrori and Van Damme 2020), and laboratory testing can rule out other conditions (Brooks et al. 2000).

Several diseases share similarities with ALS and can cause misdiagnosis, including primary lateral sclerosis (PLS), spinal muscular atrophy (SMA), Kennedy’s disease, cervical radiculo/myelopathy, post-polio syndrome, hereditary spastic paraplegia, and plexopathy or peripheral neuropathies (Singh et al. 2018; Masrori and Van Damme 2020; de Boer et al. 2023). Early and accurate diagnosis is therefore crucial for improving prognosis.

Currently, no treatments reverse neuronal damage or cure ALS, but early symptomatic management can improve outcomes. This involves a multidisciplinary team of neurologists, pulmonologists, physiotherapists, speech therapists, nutritionists, and palliative care specialists. Treatment aims to slow disease progression and alleviate symptoms. Management focuses on supportive care, including ventilatory and nutritional support, with early percutaneous endoscopic gastrostomy (PEG) recommended (Mazzini et al. 1995; Masrori and Van Damme 2020). Pharmacologically, riluzole (a glutamate release inhibitor) and edaravone (an antioxidant) are administered (Mejzini et al. 2019). New therapies are being developed, and precision medicine approaches (e.g. antisense oligonucleotides, stem cell models, gene editing, and epigenetic therapies) show promise (Mejzini et al. 2019).

### Charcot-Marie-Tooth disease

CMT, also known as hereditary motor and sensory neuropathy (HMSN), is not a single disease, but a group of inherited neuropathies that affect peripheral nerves; it is characterised by progressive distal muscle weakness, sensory loss, and foot deformities (Sivera et al. 2013). The disease is caused by mutations in over 100 genes linked to myelin formation, axonal transport, and neuromuscular function (Lupski et al. 2010). CMT is classified into several subtypes based on inheritance patterns, nerve conduction velocity (NCV), and genetic mutations (Szigeti and Lupski 2009; Association 2025); however, the major classification principle is its pathological mechanism. *Demyelinating CMT* (including CMT4 and CMTX) is characterised by primary damage to the myelin sheath, leading to reduced NCV. *Axonal CMT* (CMT2) affects the axons directly, with relatively normal NCV but reduced action potential amplitudes due to axonal degeneration. A third, uncommon category is *intermediate CMT*, which exhibits overlapping features of both demyelinating and axonal CMT (Association 2025).

CMT1 is the most common form of CMT, with approximately half of all CMT cases falling into this category (Szigeti and Lupski 2009). It is inherited in an autosomal dominant pattern (Braathen 2012) and further subcategorised into types A-J (Association 2025), depending on the affected gene. The two most frequent mutation targets are the PMP22 (peripheral myelin protein 22*)* and *MPZ* (myelin protein zero) genes, which are crucial for myelin formation and maintenance (Krajewski et al. 2000). The most common cause of CMT is copy number alteration of the *PMP22* gene.

CMT2 is primarily caused by mutations in *MFN2* (Mitofusin-2), *RAB7*,* TRPV4*,* GARS*, and *NEFL*. Notably, CMT2 overlaps the most with ALS, and *MFN2* and *GARS* mutations are among the genes identified here (see below) as having possible roles in both conditions. Although *NEFL* is not a focus of this study, neurofilaments have emerged as important biomarkers for ALS (Cairns et al. 2004; Xu et al. 2016).

Less common subtypes include CMT4, an autosomal recessive demyelinating form of CMT. The main genes associated with this subtype are *GDAP1*,* MRMR2/13*,* and SH3TC2*, and mutations disrupt myelin formation and mitochondrial function. These mutations lead to severe neuropathy, often with early onset symptoms (Sivera et al. 2013; Association 2025). CMTX is an X-linked form of CMT that affects both sexes but tends to be more severe in males due to their single X chromosome. The gene involved is *GJB1*, which encodes connexin 32, a protein involved in regulating gap junctions in Schwann cells (Association 2025). Earlier studies have documented possible co-occurrence or overlapping pathogenesis between ALS and CMTX1 (Feng et al. 2018).

Other rare forms of CMT include:

- distal hereditary motor neuropathy (dHMN): only affects motor nerves and spares sensory nerves, leading to progressive distal limb muscle weakness and atrophy (Bansagi et al. 2017).
- hereditary sensory and autonomic neuropathy (HSAN): involves sensory and autonomic nerves, leading to sensory loss, pain sensitivity, and autonomic dysfunction (Ma et al. 2023).

Although CMT is a group of rare disorders, it is the most common human hereditary neuromuscular disorder, affecting approximately 1 in 2,500 individuals worldwide (Szigeti and Lupski 2009; Ma et al. 2023). Regional variation in prevalence estimates exists, ranging from 9.7 per 100 000 people in Serbia to 82.3 per 100 000 in Norway (Ma et al. 2023). This could be partly due to differences in diagnostic methods, possible founder effects, and genetic screening availability (Braathen 2012; Ma et al. 2023). CMT1 is the most prevalent form, accounting for ~ 50% of cases (Ma et al. 2023), followed by CMT2, accounting for ~ 30% of cases. In Norway, CMT1 and CMT2 have been estimated to have roughly the same prevalence (48.2% and 49.4%, respectively) (Braathen 2012; Ma et al. 2023). CMTX accounts for 5–13% of cases. Given the chronic and hereditary nature of CMT, its incidence rates are less frequently reported.

Over 100 genes have been linked to CMT, affecting Schwann cells and myelination, axonal transport, mitochondrial function, and synaptic functions. The most commonly affected CMT genes are *PMP22*, *MPZ*, *MFN2*, and *GJB1*. Table 2 provides a brief overview of the most common CMT genetic associations and pathological mechanisms.

**Table 2:** Key genetic associations in CMT. The role of MFN2 is discussed later

Gene	Function	Pathology	Associated diseases	OMIM entry
*PMP22* (Li et al. 2013)	Myelin formation and maintenance, Schwann cell differentiation, intracellular trafficking	Duplication/deletion causes demyelination, Schwann cell dysfunction, and axonal degeneration	CMT1A	*601,097
			CMT1E	
*MPZ* **(**Su et al. 1993)	Myelin compaction, cell-cell adhesion, regulation of apoptosis	Protein misfolding, demyelination, axonal damage	CMT1B	*159,440
			CMT2I	
			CMT2J	
*GDAP1* (Cuesta et al. 2002)	Mitochondrial fission and fusion, cellular signaling and lipid metabolism	Mitochondrial dysfunction and axonal damage	CMT2B3 CMT2K CMT4A	*606,598
*GJB1* (Ionasescu et al. 1994)	Cell communication through the formation of gap junctions	Impaired Schwann cell communication	CMT1X/X1	*304,040
*NEFL* (Ismailov et al. 2001)	Structural support and axonal transport	Neurofilament accumulation, cytoskeletal instability, axonal degeneration	CMT1F	*162,280
			CMT2B5	
			CMT2E	

CMT exhibits varying clinical severity, onset, and progression between individuals. Many patients develop symptoms in early life, late adolescence, or early adulthood (Szigeti and Lupski 2009). The symptoms progress slowly, but some subtypes (CMT4 and CMT3) are severe from childhood (Szigeti and Lupski 2009; Sivera et al. 2013). An early sign of CMT is distal muscle weakness, particularly in the lower limbs. This weakness leads to foot drop, making walking difficult. To compensate, patients develop steppage gait, lifting their legs to prevent tripping. Patients also develop foot deformities, such as high-arched feet (*pes cavus*) and hammer toes, due to an imbalance in muscle innervation. As the disease advances, weakness progresses proximally, affecting the hands and forearms (Szigeti and Lupski 2009). This leads to difficulties in performing daily tasks, such as buttoning clothes. In severe cases, intrinsic hand muscle atrophy leads to claw-like finger deformities (McCorquodale et al. 2016).

Sensory symptoms affect the distal extremities and progress in a length-dependent pattern. Loss of proprioception disrupts limb position sensing, causing stumbling and balance problems. Reduced pain and temperature sensation increases risk of injuries and ulcers. Some patients experience paraesthesia, including tingling, numbness, and burning pain, especially in axonal CMT2 subtypes (Szigeti and Lupski 2009; McCorquodale et al. 2016). Although CMT primarily affects the peripheral nervous system (PNS), musculoskeletal complications are common. Scoliosis and hip dysplasia occur more frequently in early-onset CMT and may require surgery (Sivera et al. 2013; McCorquodale et al. 2016). Patients may experience muscle cramps and tremors (McCorquodale et al. 2016).

CMT diagnosis relies on clinical features, electrophysiological and genetic testing, and sometimes sural nerve biopsy. Electrophysiological testing distinguishes demyelinating CMT (reduced NCV) from axonal CMT (normal/slightly reduced NCV) (Szigeti and Lupski 2009). Genetic testing panels targeting mutations can confirm CMT diagnosis and allow subtype classification, which is important for prognosis and family planning (Szigeti and Lupski 2009; Braathen 2012; McCorquodale et al. 2016).

CMT exhibits clinical similarity with several other inherited or acquired neuropathies (diabetic polyneuropathies, chronic inflammatory demyelinating polyneuropathies [CIDP]), as well as distal myopathies; even early ALS stages may resemble CMT (Pareyson and Marchesi 2009; Braathen 2012; Li et al. 2013).

Similarly to ALS, there is no cure for CMT. The goal of current treatments is to manage the symptoms and prevent complications. The treatment approaches include supportive care (physical therapy, orthotic devices, occupational therapy, pain management (neuropathic pain drugs, muscle relaxants), and surgical intervention (foot surgery for severe *pes cavus* and hammer toes) (Szigeti and Lupski 2009; McCorquodale et al. 2016).

#### Summary of general disease features between ALS and CMT

ALS and CMT are neurological disorders that share similarities while remaining distinct in their underlying pathophysiology, progression, and clinical impact (Table 3). ALS is rapidly progressive and fatal, affecting both UMNs and LMNs within the central and peripheral nervous systems (CNS, PNS), whereas CMT is slowly progressive and primarily affects peripheral nerves. The genetic differences between ALS and CMT concern in both cases rarely affected genes, which however point towards similar cellular pathways.

**Table 3:** Comparison of the key features of ALS and CMT

Feature	ALS	CMT
Definition	Progressive neurodegenerative disorder affecting UMN and LMN, leading to muscle weakness, paralysis and respiratory failure	A heterogeneous group of inherited neuropathies affecting peripheral nerves, causing progressive distal muscle weakness, sensory loss and foot deformities
Primary neuron affected	Motor neurons (CNS and PNS)	Peripheral nerves
Onset	Mid – late adulthood (40–65 years)	Childhood or early adulthood
Key symptoms	Progressive muscle weakness, fasciculations, spasticity, dysarthria, respiratory failure	Distal muscle atrophy, sensory deficits, foot deformities, hyporeflexia
Progression	Rapid (death in 2–5 years)	Slow, chronic
Epidemiology	1–2 per 100 000 individuals annually, lifetime risk of 1 in 400	Most common inherited neuropathies (1 in 2500 individuals worldwide)
Etiology and pathophysiology	Motor neuron degeneration, oxidative stress, glutamate excitotoxicity and aggregation of misfolded proteins (e.g. TDP-43 and SOD1)	Mutations affecting myelin (CMT1, CMT, CMTX) or axonal function (CMT2), leading to impaired nerve conduction, axonal degeneration, and progressive neuropathy.
Diagnostics	Clinical criteria (El Escorial), EMG, exclusion of mimicking conditions. Genetic testing may confirm fALS	Clinical evaluation, electrophysiological studies, genetic testing and nerve biopsy
Differential diagnosis and associated disorders	SMA, PLS, myasthenia gravis	Hereditary neuropathies, distal myopathies, diabetic neuropathies
Management	Supportive care, ventilatory support, symptomatic treatment (riluzole and edaravone)	Physiotherapy, orthotic support, pain management, and if necessary, surgery

#### Insights into common target genes and molecular pathways

Despite advances in research, gaps remain in understanding the potentially shared genetic and molecular mechanisms between ALS and CMT, and the clinical implications of these overlaps have not been extensively studied. Investigating possibly common molecular pathogenic mechanisms in ALS and CMT is crucial for advancing diagnostics and disease classification; such research could improve genetic counselling and risk assessment in affected families. Genetic testing for genes implicted in both ALS and CMT could help with an early, accurate diagnosis.

**Fig. 1:**
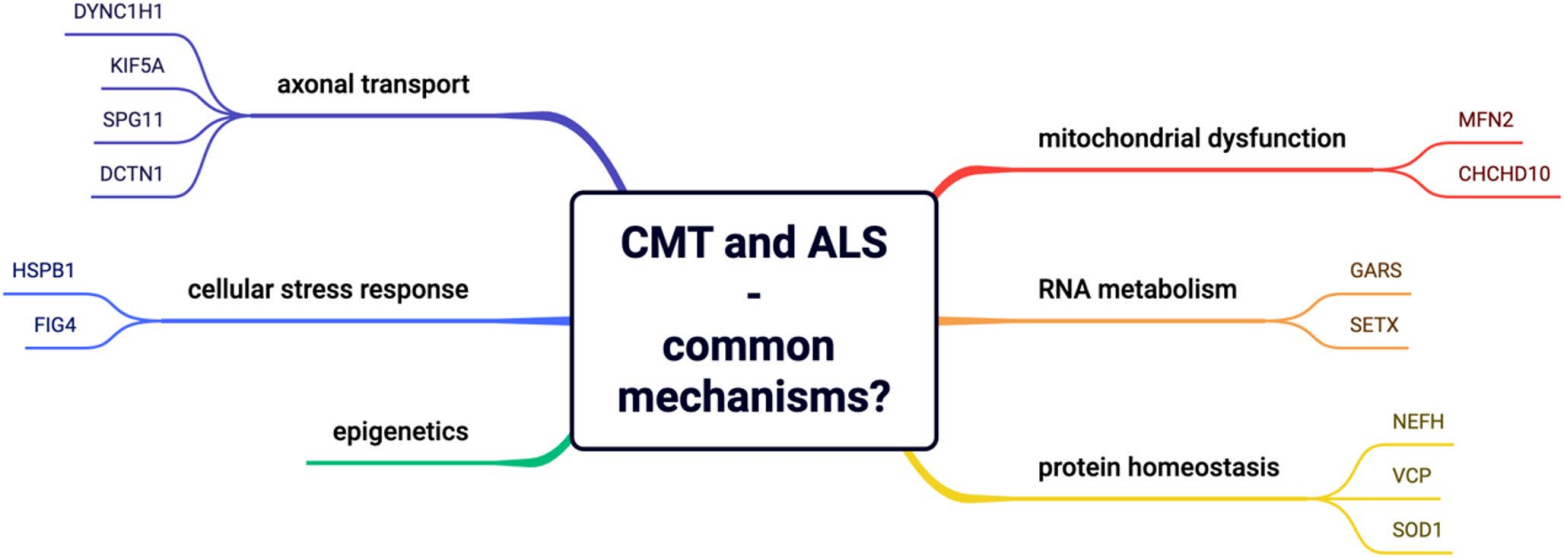
Overview of the putative common molecular mechanisms of ALS and CMT. The genes linked to a possible mechanism, with evidence of linkage to both diseases, are shown

We aimed to identify shared affected genes between fALS and CMT, focusing on possibly overlapping mechanisms, including dysfunctional axonal transport, protein aggregation, disruption of RNA metabolism, aberrant cellular stress response, and mitochondrial dysfunction. Additionally, we discuss the role of epigenetic modifiers, such as DNA methylation and histone modifications, in disease manifestation and progression. Figure 1 shows an overview of the topics discussed and possible common molecular denominators for ALS and CMT.

**Fig. 2:**
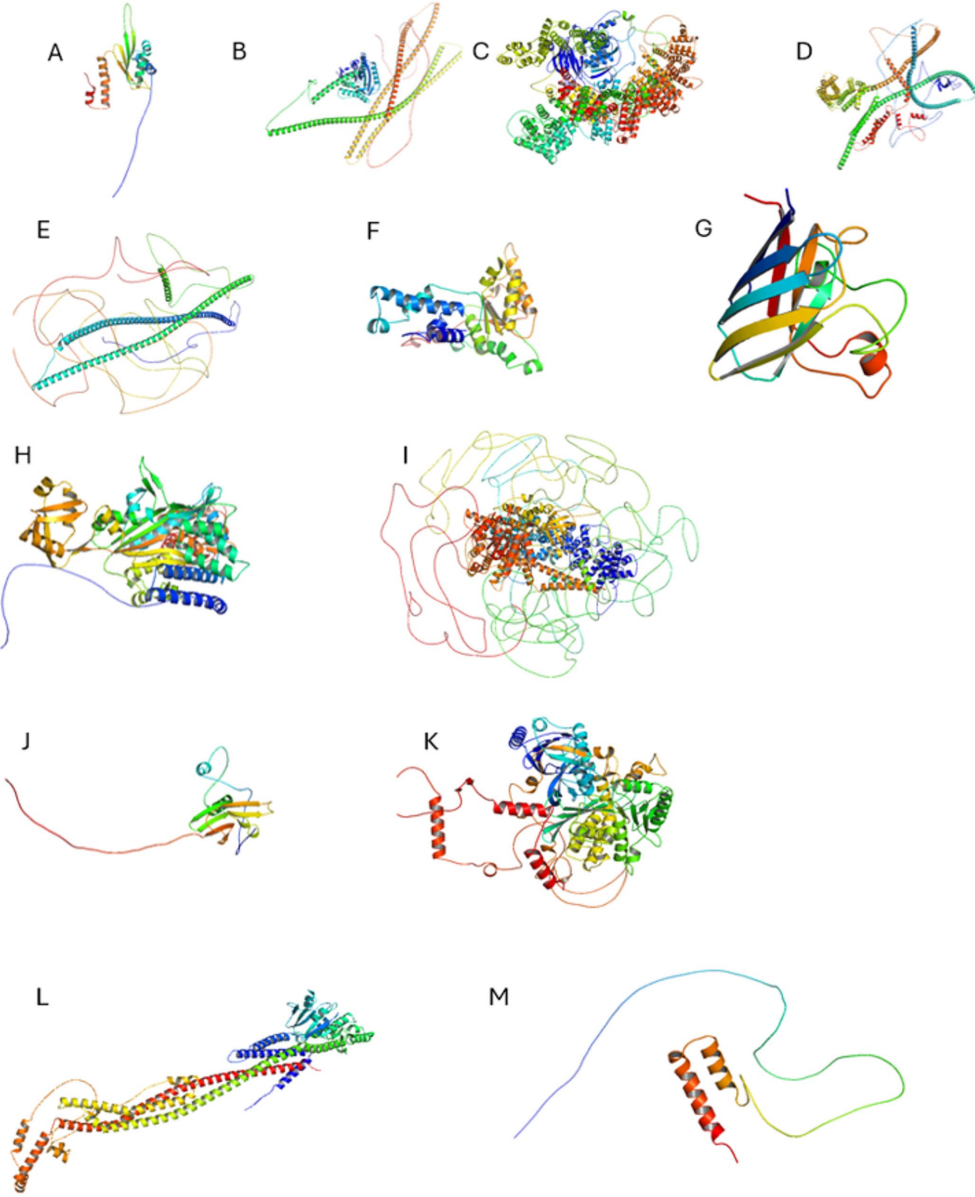
Predicted protein structures generated using AI-based modelling (AlphaFold3). The structures show the wide diversity of possible protein targets in fALS and CMT. **A** DYNC1H1. **B)** KIF5A. **C** spatacsin. **D** DCTN1. **E** NEFH. **F** VCP. **G** SOD1. **H** GARS1. **I** senataxin. **J** HSPB1. **K** FIG4. **L** MFN2. **M** CHCHD10

We identified 13 genes associated with both fALS and CMT in the literature, highlighting putative molecular and cellular pathways that contribute to certain rare subtypes of both diseases. Structural models for each protein are shown in Fig. 2, highlighting the large diversity of protein structure types involved. Table 4 summarises the key features of the genes discussed in this work. The genetic commonalities between ALS and CMT identified here suggest diverse, complex pathogenic mechanisms, which can be roughly categorised based on their predicted molecular-level impact. Mutations in *DYNC1H1*,* KIF5A*,* SPG11*, and *DCTN1* likely affect axonal transport. *VCP*,* NEFH*, and *SOD1* mutations disrupt protein homeostasis and aggregation. *GARS* and *SETX* mutations affect RNA metabolism, while those in *HSPB1* and *FIG4* compromise cellular stress responses. Finally, *MFN2* and *CHCHD10* mutations are associated with mitochondrial dysfunction. Below, the identified genes that are mutated in rare cases of both CMT and fALS are discussed in the context of possible pathological mechanisms at both the molecular and cellular levels.

**Table 4:** Summary of possible common gene targets involved in fALS and CMT

Gene	Protein function	Pathology	Associated diseases	OMIM
*DYNC1H1* (Weedon et al. 2011; Martin et al. 2020)	Retrograde axonal transport	Mutations alter dynein processivity and interaction with microtubules, leading to impaired transport	CMT2O, SMA, and CDCBM13.	*600,112
*KIF5A* (Nam et al. 2018a; Martin et al. 2020; Cozzi et al. 2024)	Anterograde axonal transport	Misregulation leads to toxic aggregation and disruptions in mitochondrial transport	CMT2, ALS, SPG10, and neonatal intractable myoclonus (NEIMY)	*602,821
*SPG11* (Kenna et al. 2013; Montecchiani et al. 2016; Martin et al. 2020)	Lysosomal trafficking and axonal maintenance	Truncating and missense mutations lead to lysosomal dysfunction, impairing autophagy	ALS5, CMT2X, and HSP	*610,844
*DCTN1* (Vilariño-Güell et al. 2009; Nam et al. 2016; Martin et al. 2020)	Retrograde axonal transport and autophagy trafficking	Mutations impair DCTN1 interactions with dynein, leading to protein accumulation and cytoskeletal dysfunction	Perry syndrome, ALS, and dHMN7B	*601,143
*NEFH* (Jacquier et al. 2017; Nam and Choi 2019)	Axonal transport and stability	Mutations introduce CAE, leading to protein aggregation	ALS and CMT2CC	*162,230
*VCP* *(*Gonzalez et al. 2014; Scarian et al. 2022)	Protein degradation, autophagy, mitochondrial quality control	Mutations lead to misfolding, defective autophagy, oxidative stress and neuronal death	CMT2Y, ALS6-FTD, and IBMPFD	*601,023
*SOD1* (Peggion et al. 2022)	Antioxidant enzyme, protecting against oxidative stress	Aggregated SOD1 spreads between cells in a prion-like manner. Often found in mitochondria, affecting energy production	ALS1 and STAHP	*147,450
*GARS* (Antonellis et al. 2006; Boczonadi et al. 2018; Martin et al. 2020)	Protein synthesis	Mutations impair tRNA charging, leading to protein synthesis defects and toxic aggregates	CMT2D, ALS, and dHMN	*600,287
*SETX* (Tsui et al. 2023)	Transcriptional regulation and DNA repair	Mutations disrupt senetaxin functions and contribute neurodegeneration	ALS4, CMT, and AOA2	*608,465
*HSPB1* (Houlden et al. 2008)	Proteostasis and cytoskeletal integrity	Mutations alter chaperone activity, causing protein aggregation and axonal degeneration	CMT2F, dHMN, and recently ALS	*602,195
*FIG4* (Chow et al. 2009)	Lipid metabolism, phosphoinositide signaling and lysosomal trafficking	Mutations cause disruptions in axonal transport, lysosomal dysfunction and have secondary effects on mitochondria	CMT4J, ALS11, bilateral temporooccipital polymicrogyria and Yunis-Varon syndrome	*609,390
*MFN2* (Kijima et al. 2005; Abati et al. 2024)	Mitochondrial fusion and axonal transport	Defective mitochondrial transport leads to axonal degeneration	CMT2A, ALS, ALS-FTD, HMSN VI, optic atrophy	*608,507
*CHCHD10* (Bannwarth et al. 2014; Auranen et al. 2015)	Mitochondrial cristae structure and oxidative phosphorylation	Mutations lead to mitochondrial fragmentation, decreased oxidative phosphorylation and instability of the mitochondrial genome	ALS, FTD, CMT2, mitochondrial myopathy, and SMAJ	*615,903

#### Axonal transport dysfunction

Dysfunction in axonal transport is common in both ALS and CMT, and many known mutations linked to these diseases affect intracellular transport. Mutations in *DYNC1H1* impair dynein function, affecting retrograde transport, which is crucial for organelle recycling and overall cell maintenance (Hafezparast et al. 2003; Weedon et al. 2011). *KIF5A* mutations impair anterograde transport, impacting key functions such as synaptic signalling and mitochondrial trafficking (Hirokawa and Tanaka 2015). Notably, differences in the effects of *KIF5A* mutations (toxic aggregation in ALS vs. transport deficits in CMT) contribute to disease-specific manifestations (He et al. 2020; Cozzi et al. 2024). While *DCTN1* mutations are primarily associated with ALS and not considered causative for CMT, they are associated with conditions (e.g. dHMN7B) that lie on the spectrum of distal motor neuropathies with overlapping features (Vilariño-Güell et al. 2009; Martin et al. 2020). Mutations in *SPG11* lead to loss of spatacsin function, impairing lysosomal activity and cargo transport. Both conditions exhibit axonal swelling, mitochondrial clustering, and impaired endosomal trafficking, underscoring the critical role of spatacsin in vesicle transport (Gentile et al. 2019). The following sections discuss the roles of the four genes (*DYNC1H1*,* KIF5A*,* SPG11*, and *DCTN1*) in ALS and CMT.

#### DYNC1H1

*DYNC1H1* encodes cytoplasmic dynein 1 heavy chain 1, a motor protein critical for retrograde axonal transport of organelles, vesicles, and protein complexes along microtubules (Weedon et al. 2011; Martin et al. 2020). Mutations in *DYNC1H1* disrupt retrograde transport and often affect the domains responsible for dimerisation, motor activity, or interactions with intermediate and light chains, thereby altering dynein processivity and its interactions with microtubules. These defects in dynein function reduce retrograde transport efficiency and result in axonal degeneration, particularly in sensory and motor neurons with long axons (Weedon et al. 2011; Martin et al. 2020). *DYNC1H1* is associated with a spectrum of neurological and neuromuscular diseases, including CMT2O, spinal muscular atrophy (SMA), and complex cortical dysplasia with other brain malformations-13 (CDCBM13) (Martin et al. 2020).

Variants in *DYNC1H1*, such as *c.4106 A > G (p.Gln1369Arg)*, affect dynein stability and retrograde axonal transport. These defects manifest as impaired protein recycling, disrupted neuronal migration, and motor neuron degeneration. The *c.4106 A > G* variant has been identified in a patient with ALS-FTD spectrum disorder (Mentis et al. 2022).

In a large pedigree with dominant axonal CMT2, *c.917 A > G (p.His306Arg)* was identified (Weedon et al. 2011). This variant in the dimerisation domain disrupts retrograde axonal transport.

Impaired dynein function is a central feature of both ALS and CMT, disrupting the transport of vital cargo and contributing to neuronal degeneration. In ALS, motor neuron degeneration arises from defective retrograde transport mediated by *DYNC1H1* mutations, while in CMT, similar mutations lead to axonal degeneration in sensory and motor neurons. The vulnerability of long axons in both diseases underscores the critical role of axonal transport in maintaining neuronal integrity (Weedon et al. 2011; Mentis et al. 2022).

#### KIF5A

*KIF5A* encodes the kinesin family member 5 A, a neuron-specific kinesin motor critical for anterograde axonal transport (Hirokawa and Tanaka 2015), which facilitates the movement of organelles, proteins, and RNA along axonal microtubules, ensuring neuronal function (Martin et al. 2020; ScienceDirect 2023). Mutations in *KIF5A* lead to neuronal degeneration. Such dysfunctions are implicated in several disorders, including ALS, CMT2, spastic paraplegia (SPG10), and neonatal intractable myoclonus (NEIMY) (Nam et al. 2018a; Martin et al. 2020; Cozzi et al. 2024).

In ALS, a frameshift mutation *(c.2993_3020del*, p.Asn999Valfs*40) within the KIF5A tail domain disrupts autoinhibition, leading to constitutive activation of KIF5A. This misregulation results in toxic aggregation, whereby mutant KIF5A sequesters wild-type KIF5A and impairs proteostasis pathways, such as the ubiquitin-proteasome system. Axonal transport deficits and mitochondrial dysfunction manifest as late-onset ALS with rapid progression and early mortality (He et al. 2020; Cozzi et al. 2024).

CMT patients with *KIF5A* mutations experience length-dependent axonal neuropathy with sensory deficits and motor impairment. In severe cases, symptoms include pyramidal signs, deafness, and cognitive impairment (He et al. 2020; Cozzi et al. 2024), and mutations (*c.2591 C > T*, p.Arg864* and *c.610 C > T p.Arg204Trp*) disrupt axonal transport. The (c.2591 C > T) nonsense mutation creates a truncated, non-functional protein (Cozzi et al. 2024). The (c.610 C > T) missense mutation impairs motor domain interactions with microtubules (He et al. 2020). These mutations cause loss of function, affecting mitochondrial transport and neuronal signalling (Nam et al. 2018a; He et al. 2020).

Overall, mutations in *KIF5A* result in distinct mechanisms in ALS and CMT (Table 5). In ALS, frameshift mutations lead to a toxic gain of function involving protein aggregation and disruption of proteostasis (Cozzi et al. 2024). In contrast, CMT mutations cause both gain and loss of function effects, impairing autoinhibition and mitochondrial transport and leading to axonal neuropathy (He et al. 2020; Cozzi et al. 2024). Thus, mitochondrial dysfunction and axonal transport disruption may be shared pathological features (Martin et al. 2020; Berth and Lloyd 2023; Cozzi et al. 2024).

**Table 5:** *KIF5A* mutations in ALS and CMT

Feature	ALS (e.g. c.2993_3020del, *p*.Asn999Valfs*40)	CMT (e.g. c.2591 C > T, *p*.Arg864* and c.610 C > T, *p*.Arg204Trp)
Mutation type	Frameshift	Nonsense/Missense
Protein impact	Toxic gain-of-function	Loss of function
Mechanism	Aggregation, proteostasis disruption	Impaired mitochondrial transport
Pathological consequences	Rapid motor neuron degeneration	Peripheral axonal neuropathy

#### SPG11

*SPG11* (spastic paraplegia 11) encodes spatacsin, a protein integral to lysosomal trafficking, axonal maintenance, and neuronal survival. Spatacsin functions in endo-lysosomal sorting and vesicle trafficking, which are essential for axonal integrity and neuronal function (Montecchiani et al. 2016; Gentile et al. 2019; Martin et al. 2020). Mutations in *SPG11* are linked to neurodegenerative diseases, including juvenile-onset autosomal recessive ALS (ARJALS), autosomal recessive axonal CMT2, and autosomal recessive hereditary spastic paraplegia (ARHSP) (Abdel Aleem et al. 2011). SPG11 mutations, often truncating or missense, lead to loss of spatacsin function, impairing lysosomal function and cargo transport. Dysregulated autophagy exacerbates neuronal stress and degeneration, affecting both central and peripheral neurons (Kenna et al. 2013; Montecchiani et al. 2016; Martin et al. 2020).

*SPG11* mutations in juvenile ALS present as slowly progressive UMN and LMN, such as spasticity and distal muscle wasting. Post-mortem analyses show axonal degeneration and typical ALS neuropathology, with motor neuron degeneration and axonal swelling (Orlacchio et al. 2010). *SPG11* mutations display phenotypic variability, from ARJALS to ARHSP. The mutation *c.7155T *> G (p.Tyr2385*) results in truncated spatacsin, causing severe motor neuron disease with overlapping ALS and HSP features (Iskender et al. 2015). Such mutations disrupt the ability of spatacsin to facilitate vesicle trafficking (Daoud et al. 2012; Iskender et al. 2015).

CMT2 patients with *SPG11* mutations exhibit early-onset symptoms, including distal motor and sensory neuropathy, and are often misdiagnosed as HSP due to overlapping clinical features. In autosomal recessive axonal CMT, specific mutations, such as c.704_705delAT (p.His235Argfs*12) and c.2678G > A (p.Trp893*), were identified (Montecchiani et al. 2016). This leads to the disruption of spatacsin function and progressive axonal degeneration (Montecchiani et al. 2016).

*SPG11* mutations illustrate a possible link between ALS and CMT through shared mechanisms, particularly disruption of axonal transport and autophagy. Both conditions exhibit axonal swelling, mitochondrial clustering, and impaired endosomal trafficking, underscoring the critical role of spatacsin in vesicle transport (Montecchiani et al. 2016; Gentile et al. 2019; Martin et al. 2020).

#### DCTN1

*DCTN1* encodes p150(Glued), the largest subunit of the dynactin complex, important for retrograde axonal transport and autophagosomal trafficking. The complex links cargo to dynein motors and stabilises microtubule interactions. *DCTN1* mutations affect the microtubule-binding domain of dynactin and interactions with dynein, leading to impaired retrograde transport, cargo accumulation in axons, and disruption of the cytoskeleton. *DCTN1* mutations have been implicated in Perry syndrome, ALS, and dHMN7B (Vilariño-Güell et al. 2009; Nam et al. 2016; Martin et al. 2020). Notably, Perry syndrome shares features with ALS, including parkinsonism, depression, and TDP-43 inclusions (Vilariño-Güell et al. 2009).

Regarding ALS, disruption of p150(Glued) leads to TDP-43-positive inclusions, contributing to cytotoxicity (Martin et al. 2020). Examples of mutations found in ALS include *c.175G > A (p.Gly59Ser)* (Münch et al. 2004; Vilariño-Güell et al. 2009), c.3746 C > T (*p.Thr1249Ile*) (an sALS case), *c.1712T > C (p.Met571Thr)* (an fALS case), and *c.2353 C > T (p.Arg785Trp)* (found in two fALS cases and two unaffected relatives, indicating variable penetrance) (Münch et al. 2004). While *DCTN1* mutations have been identified in patients with ALS, there is no conclusive association between *DCTN1* mutations and ALS, Parkinson’s disease, or frontotemporal lobar degeneration (FTLD) (Münch et al. 2004; Vilariño-Güell et al. 2009).

DCTN1 mutations are not a primary cause of classical CMT; they are associated with conditions on the spectrum of distal motor neuropathies with overlapping features. The overlap between dHMN7B and CMT could explain why some patients initially diagnosed with CMT may actually have dHMN7B (Martin et al. 2020). A *DCTN1* mutation *(c.1685 A > G*,* p.Glu562Gly)* was identified in a patient with dHMN7B, which could contribute to CMT-like symptoms (Nam et al. 2016). Direct evidence linking *DCTN1* mutations to CMT is limited, and further studies are necessary (Martin et al. 2020).

#### Protein homeostasis and aggregation

Protein homeostasis is important for neuronal function. In both ALS and CMT, mutations in genes involved in cellular homeostasis and proteostasis (*NEFH*,* VCP*, and *SOD1*) may lead to protein misfolding and aggregation and neuronal toxicity. *SOD1* mutations cause oxidative stress through protein aggregation; they represent a known ALS cause, but are less frequently linked to CMT (Bunton-Stasyshyn et al. 2015; Luo et al. 2022). *NEFH* mutations contribute to neurotoxicity *via* protein aggregation. In CMT, neurofilament defects cause failure to maintain axonal stability and result in distal axon degeneration (Jacquier et al. 2017), while in ALS, neurofilament accumulation and cytoskeletal collapse affect motor neuron axons (Lin et al. 2021). Long axons, like those in corticospinal tracts (ALS) and peripheral nerves (CMT), are especially vulnerable to this type of defect. *VCP* mutations impair autophagy and proteostasis, disrupt mitochondrial quality control, and cause axonal degeneration. *VCP*, like *SOD1*, is classically associated with ALS, but a missense mutation was identified in a family with autosomal dominant CMT2 (Gonzalez et al. 2014). The following section discusses the roles of these genes in ALS and CMT.

#### NEFH

*NEFH* (neurofilament heavy chain) encodes the heavy subunit of neurofilaments, intermediate filaments essential for maintaining neuronal cytoskeletal integrity and regulating axonal diameter in large, myelinated axons. This regulation influences axonal transport and nerve conduction velocity (Nam and Choi 2019; Martin et al. 2020). Neurofilaments provide a framework for organelle and vesicle trafficking in both motor and sensory axons. Frameshift mutations in *NEFH* result in cryptic amyloidogenic elements (CAE), leading to protein aggregation (Jacquier et al. 2017). These aggregates disrupt axonal transport, resist autophagic degradation, and cause neuronal apoptosis. *NEFH* has been associated with both ALS and CMT2CC (Jacquier et al. 2017; Nam and Choi 2019).

Rare ALS cases have been linked to *NEFH* mutations, which alter transcription patterns, causing abnormal protein interactions and disrupting the neuronal cytoskeleton (Jacquier et al. 2017; Martin et al. 2020). Dominant missense or truncating mutations affecting the tail domain of NEFH have been implicated in ALS (Marriott et al. 2024). Aggregates of mutant NEFH in motor neurons of ALS patients cause cytoskeletal disruption, impair axonal transport, and affect mitochondrial function (Lin et al. 2021).

*NEFH* mutations, particularly frameshift or missense, cause CMT2CC, a rare autosomal dominant axonal neuropathy. These mutations produce truncated proteins containing CAE, which aggregate and disrupt neurofilament assembly and transport (Jacquier et al. 2017; Nam and Choi 2019). Patients with CMT2CC show early-onset (childhood to late adulthood) motor deficits and progressive sensory impairment (Jacquier et al. 2017; Martin et al. 2020). Like ALS, disrupted neurofilament assembly and axonal transport lead to axonal degeneration. Distal motor and sensory axons are vulnerable due to their length-dependent reliance on neurofilament structures. Loss of neurofilament integrity reduces axonal diameter, impairing signal transmission and causing CMT symptoms (Jacquier et al. 2017; Martin et al. 2020).

ALS and CMT subtypes present disrupted axonal transport and cytoskeletal defects, and *NEFH* mutations cause neurotoxicity through aggregate formation. In CMT, neurofilament failure leads to distal axon degeneration, while in ALS, neurofilament accumulation affects motor neuron axons. Again, long axons in corticospinal tracts (ALS) and peripheral nerves (CMT) are particularly vulnerable.

#### VCP

*VCP* encodes the valosin-containing protein (VCP/p97) in the type II AAA (ATPase associated with diverse cellular activities) family (Yamaguchi et al. 2021a; Scarian et al. 2022). VCP is crucial for maintaining cellular protein homeostasis, regulating protein degradation through both the ubiquitin-proteasome and autophagic pathways. VCP is also involved in lysosomal clearance and mitochondrial quality control (Gonzalez et al. 2014; Martin et al. 2020), as well as endoplasmic reticulum-associated degradation (ERAD), cell cycle regulation, and stress response (Martin et al. 2020; Korb et al. 2022). *VCP* mutations disrupt autophagic flux and proteasomal degradation, causing misfolded protein accumulation (Gonzalez et al. 2014; Martin et al. 2020). They also lead to defective mitochondrial quality control, causing oxidative stress and neuronal death (Gentile et al. 2019). *VCP* has been associated with ALS6-FTD, CMT2Y, and inclusion body myopathy with Paget’s disease of bone and frontotemporal dementia (IBMPFD), as well as rare cases of HSP and cardiomyopathy (Korb et al. 2022).

*VCP* mutations were among the first to be linked to the ALS-FTD spectrum, characterised by motor neuron degeneration and cognitive impairment. Mutations in *VCP* account for 1–2% of fALS (Gentile et al. 2019; Scarian et al. 2022) and lead to dysregulation of protein homeostasis with protein aggregation, causing cytosolic TDP-43 aggregation, followed by ER stress and cell death. TDP-43 inclusions and mislocalisation are observed in ALS (and CMT), contributing to neurotoxicity (Scarian et al. 2022). The mutation *c.464G > A (p.Arg155His)* is strongly associated with ALS and impairs VCP function in protein degradation (Martin et al. 2020), leading to increased cytosolic TDP-43 aggregation and cell death (Scarian et al. 2022).

Missense mutations in *VCP* have been implicated in autosomal-dominant CMT2. The mutation *c.553G > A (p.Glu185Lys)* was identified in a family with CMT2 (Gonzalez et al. 2014). This mutation disrupts autophagic flux, causing immature autophagosome accumulation and axonal degeneration (Gonzalez et al. 2014). Patients with *VCP*-related CMT2 show a length-dependent pattern of axonal degeneration, with sensory and motor nerves affected in distal regions (Gonzalez et al. 2014).

Similar pathophysiological mechanisms may be driven by *VCP* mutations in ALS and CMT (Martin et al. 2020); a common feature is disruption of autophagy and proteostasis. *VCP* mutations impair clearance of misfolded proteins and dysfunctional organelles, causing cellular toxicity. Mutations compromise mitochondrial quality control, leading to oxidative stress (Gentile et al. 2019). Although TDP-43 proteinopathy is a distinguishing feature of ALS, it is not typically associated with CMT. The presence of autophagic and proteasomal disruptions highlights putative therapeutic targets (Gonzalez et al. 2014; Scarian et al. 2022).

#### SOD1

*SOD1* (superoxide dismutase 1) encodes a cytosolic metalloenzyme critical for cellular defense against oxidative stress. SOD1 protects motor neurons, susceptible to oxidative damage due to their high metabolic activity and extensive axonal projections (Bunton-Stasyshyn et al. 2015). Misfolded SOD1 aggregates can spread between cells *via* extracellular vesicles in a prion-like manner (Bunton-Stasyshyn et al. 2015), contributing to the spread of the disease within the CNS. Mutant SOD1 also abnormally localises to mitochondria, disrupting energy metabolism. Combined with the elevated production of reactive oxygen species (ROS), this damages mitochondrial DNA and proteins (Peggion et al. 2022).

The pathological effects of mutant SOD1 extend beyond motor neurons. In glial cells, mutant SOD1 disrupts neuroprotective signalling, and in skeletal muscle, it induces oxidative stress and impairs neuromuscular junctions (Peggion et al. 2022). Mutant SOD1 damages axonal transport mechanisms and activates microglia, resulting in chronic neuroinflammation, a characteristic of progressive motor neuron loss in ALS (López-Pingarrón et al. 2023). *SOD1*-associated diseases include ALS1 and progressive spastic tetraplegia and axial hypotonia (STAHP).

Mutations in *SOD1* are a well-established cause of ALS, first identified in fALS in 1993 (Siddique and Ajroud-Driss 2011). *SOD1* mutations account for approximately 20% of fALS and 2–7% of sALS cases. Over 180 mutations have been identified, most of which lead to toxic gain of function, such as protein misfolding, aggregation, and disruption of cellular redox homeostasis (Peggion et al. 2022). Patients with *SOD1* mutations often experience early, aggressive motor neuron degeneration, while disease severity and onset vary depending on the specific mutation. For example, *c.11 C > T (p.Ala4Val)* (Peggion et al. 2022) is associated with rapid disease progression and shorter survival, whereas *c.269 A > C (p.Asp90Ala)* is associated with milder disease and slower progression (Peggion et al. 2022).

A case report suggested that *SOD1* mutations may be implicated in certain forms of HMSN, specifically the axonal type (Luo et al. 2022). The authors described a 50-year-old woman who presented with progressive lower extremity weakness and muscle atrophy and was subsequently diagnosed with CMT. EMG findings revealed motor nerve conduction abnormalities, consistent with hereditary neuropathy. Genetic testing identified an *SOD1* mutation *(c.140 A > G*,* p.His47Arg)* in both a CMT patient and her daughter. While this mutation is commonly associated with ALS, it was implicated in this instance as a possible cause of CMT. The report hypothesises that *SOD1* mutations may serve as a shared pathogenic link between CMT and ALS by contributing to oxidative stress and neurodegeneration (Luo et al. 2022).

*SOD1* mutations are a well-established cause of ALS but much less frequently linked to CMT (Høyer et al. 2014). Shared pathways and even common mutations highlight the importance of oxidative stress, mitochondrial dysfunction, and proteinopathy in degeneration of motor and sensory neurons. The linking of *SOD1* to CMT highlights the need for further investigation into the role of *SOD1* mutations in peripheral neuropathies and their potential connection to neurodegeneration mechanisms (Østern et al. 2012; Høyer et al. 2014; Luo et al. 2022).

#### RNA metabolism and translation disruption

Proper RNA metabolism and protein translation are essential for regulating gene expression and cellular homeostasis. Mutations in genes involved in these processes lead to dysregulated RNA processing and the impairment of protein synthesis. Defective RNA processing contributes to ALS, while emerging evidence suggests similar mechanisms in CMT (Liao et al. 2015). Mutations in *GARS* and *SETX* disrupt RNA metabolism, impairing neuronal endurance to cellular stress. *GARS* mutations impair the ability of the GARS protein to charge tRNA with glycine, disrupting protein synthesis (Antonellis et al. 2006). *GARS* mutations are linked to rare fALS cases of progressive motor neuron degeneration (Corcia et al. 2019). In CMT, specific mutations result in severe early-onset axonal CMT. Mutations in *SETX* lead to transcriptional stress, unresolved R-loops, and impaired DNA damage repair (DDR) (Motley et al. 2011; Høyer et al. 2014). In ALS, mutations impair the ability of senataxin to regulate transcriptional termination and resolve R-loops, resulting in DNA damage and motor neuron degeneration (Tsui et al. 2023). The following sections discuss the roles of *GARS* and *SETX* in ALS and CMT.

#### GARS

*GARS* encodes glycyl-tRNA synthetase (GARS), which functions in maintaining protein synthesis in both cytoplasmic and mitochondrial compartments. Additionally, GARS is implicated in the maintenance of axonal integrity, particularly within motor neurons, and it is a key player in RNA metabolism and intracellular transport (Antonellis et al. 2006; Boczonadi et al. 2018; Martin et al. 2020).

Mutations impair GARS in charging tRNA with glycine. This loss-of-function mechanism affects cellular homeostasis and axonal maintenance, leading to neurodegeneration (Antonellis et al. 2006; Boczonadi et al. 2018). In addition to loss-of-function, some mutations exhibit gain-of-function toxicity, resulting in aberrant interactions with cellular components. Mutant GARS is often mislocalised within neurons, particularly in peripheral axons, leading to impaired axonal transport and toxic aggregates (Boczonadi et al. 2018; Vinogradova et al. 2021). The phenotypes of *GARS*-related diseases range from severe infantile-onset neuropathy to milder, slowly progressive forms. This variability is influenced by the specific mutations, genetic background, and environmental factors (Martin et al. 2020). Diseases associated with *GARS* include CMTD2, ALS, and dHMN (Liao et al. 2015; Corcia et al. 2019).

*GARS* mutations have been linked to rare cases of fALS, where patients present with progressive motor neuron degeneration. For example, a 70-year-old woman with fALS exhibited bulbar symptoms, such as dysarthria and dysphonia, linked to the missense mutation (*c.2155G* > *T, p.V665L*) in *GARS* (Corcia et al. 2019).

*GARS* mutations, such as *c.616G > T (p.Asp146Tyr)* and *c.875T > G (p.Met238Arg)*, result in early-onset axonal CMT (Liao et al. 2015). These mutations are associated with severe symptoms, including muscle weakness, sensory deficits, and progressive neuropathy, beginning in infancy or childhood (Liao et al. 2015). A study on a cohort of 54 patients with an unassigned axonal CMT subtype (selected from 340 unrelated CMT patients) identified these mutations as causing early and severe forms of CMT2D (Liao et al. 2015), highlighting the importance of genetic screening for early diagnosis and intervention (Motley et al. 2011).

*GARS* mutations disrupt axonal integrity in both ALS and CMT, although the affected motor neuron populations and onset patterns differ (Corcia et al. 2019; Martin et al. 2020). Disrupted ribosomal quality control, impaired RNA metabolism, and axonal transport deficits are observed, with pathophysiological overlap in the broader spectrum of *GARS*-related neurological disorders (Boczonadi et al. 2018; Martin et al. 2020). Peripheral axons are particularly sensitive to GARS dysfunction because of their high metabolic demands and reliance on efficient protein synthesis and transport (Antonellis et al. 2006).

#### SETX

*SETX* encodes senataxin, a DNA/RNA helicase crucial for genomic stability. Senataxin functions in transcriptional regulation, R-loop resolution, and DDR. It ensures proper transcriptional termination by interacting with RNA polymerase II, facilitates the repair of double-strand breaks (DSB), prevents transcription-replication conflicts, and modulates autophagy-related pathways to clear damaged cellular components (Tsui et al. 2023). Mutations in *SETX* disrupt these processes, leading to transcriptional stress, unresolved R-loops, and impaired DDR, all of which contribute to neurodegeneration. *SETX* is implicated in ALS, particularly the juvenile form ALS4, CMT, and ataxia with oculomotor apraxia type 2 (AOA2).

ALS mutations in *SETX*, such as *c.1166T > C (p.Leu389Ser)* and *c.6407G > A (p.Arg2136His)*, impair senataxin ability to regulate transcriptional termination and resolve R-loops (Bennett et al. 2013). This failure results in unresolved R-loops that stall replication forks, induce DNA damage, and disrupt transcription. *p.Leu389Ser*, for instance, promotes the formation of stress granules, accelerating motor neuron degeneration. Additionally, *SETX* mutations result in the nuclear depletion and cytoplasmic aggregation of TDP-43 (Tsui et al. 2023). Studies on mice carrying *SETX* mutations, such as *p.Leu389Ser*, have demonstrated motor neuron degeneration, progressive neuromuscular defects, and abnormal nuclear membranes (Tsui et al. 2023). ALS4 patients exhibit increased DNA damage and defective autophagy, with enhanced stress granule formation contributing to neuronal vulnerability (Drew et al. 2011; Tsui et al. 2023).

In CMT, *SETX* mutations primarily affect axonal function and myelin maintenance. *SETX* is not commonly associated with CMT, but disruptions in axonal transport and transcriptional termination cause sensory and motor deficits characteristic of CMT. Additionally, impaired regulation of RNA/DNA hybrids affects myelin homeostasis, contributing to CMT pathophysiology (Szigeti and Lupski 2009; Li et al. 2013). Clinical studies have identified *SETX* variants in CMT patients with mixed sensory and motor neuropathies. These mutations are associated with transcriptional termination defects and unresolved R-loops (Høyer et al. 2014).

In summary, there are both common features and distinctions between the roles of *SETX* in ALS and CMT. Both diseases involve unresolved R-loops, leading to transcriptional stress and DNA damage (Drew et al. 2011). Impaired DDR is another possibly shared mechanism that increases neuronal vulnerability. Protein mislocalisation, although more pronounced in ALS, also disrupts RNA metabolism and axonal transport in CMT (Høyer et al. 2014; Tsui et al. 2023).

#### Cellular stress response disruption

Neurons rely on robust stress response mechanisms to maintain cellular integrity; however, mutations in *HSPB1* and *FIG4* may disrupt these defences. ALS mutations in *HSPB1* impair the clearance of misfolded proteins, leading to increased aggregation and neuronal toxicity (Capponi et al. 2016). In CMT, *HSPB1* mutations often affect the α-crystallin domain, which is crucial for dimerisation and chaperone activity, resulting in axonal degeneration and increased vulnerability to unfolded protein stress (Houlden et al. 2008). By disrupting phosphoinositide metabolism, mutations in *FIG4* impair endosome-lysosome trafficking, causing demyelination in CMT and neuronal death in ALS (Kitani-Morii and Noto 2020; Yamaguchi et al. 2021a).

#### HSPB1

*HSPB1*, also known as *HSP27*, encodes the heat shock protein beta-1. This protein belongs to the small heat shock protein family and functions as a molecular chaperone, maintaining cellular proteostasis (Capponi et al. 2016). HSPB1 plays a role in mitigating stress responses, including oxidative stress and the unfolded protein response (Houlden et al. 2008; Capponi et al. 2016). It interacts with cytoskeletal components, such as actin and tubulin, to ensure cellular integrity under stress conditions. HSPB1 also regulates apoptosis and axonal transport mechanisms. Mutations have been linked to CMT2, dHMN, and more recently, ALS (Martin et al. 2020).

*HSPB1* is essential for protein homeostasis by preventing the aggregation of misfolded proteins and promoting their refolding or degradation. In ALS, protein aggregation is a pathological characteristic, and HSPB1 plays a key role in mitigating this process. Mutations in *HSPB1* lead to impaired clearance of misfolded proteins, resulting in increased aggregation and neuronal toxicity (Capponi et al. 2016). Dysfunctional HSPB1 compromises axonal transport by destabilising microtubules and reducing the levels of acetylated α-tubulin (Ylikallio et al. 2015; Lu et al. 2022). Several mutations in *HSPB1*, such as c.610dupG (p.Ala204Glyfs*6) and *c.570G > C (p.Gln190His)*, have been identified in patients with ALS. These mutations impair HSPB1 and result in protein aggregation (Capponi et al. 2016).

Mutations in *HSPB1* are associated with axonal forms of CMT, such as CMT2F and dHMN (Houlden et al. 2008). These mutations frequently affect the α-crystallin domain or the C-terminal region of the protein (Zhang et al. 2022), both of which are crucial for dimerisation and chaperone activity (Houlden et al. 2008; Ylikallio et al. 2015). Loss-of-function mutations disrupt cytoskeletal integrity and axonal transport, worsening the vulnerability to unfolded protein stress and leading to axonal degeneration (Ylikallio et al. 2015).

Through its roles in protein homeostasis, cytoskeletal stabilisation, and stress response pathways, HSPB1 is important for neurons and sometimes involved in the pathogenesis of both ALS and CMT. The role of *HSPB1* mutations in ALS includes the impairment of its chaperone activity, leading to toxic protein aggregation and motor neuron degeneration (Capponi et al. 2016). In CMT, *HSPB1* mutations result in progressive motor and sensory neuropathy (Zhang et al. 2022).

#### FIG4

*FIG4* (factor-induced gene 4) encodes a phosphoinositide 5-phosphatase. FIG4 is crucial for lipid metabolism, axon maintenance, phosphoinositide signalling, and regulation of endosomal-lysosomal trafficking and vesicular sorting. Mutations in *FIG4* can disrupt these processes, damaging neuronal viability by preventing the clearance of cellular debris and hindering organelle trafficking (Martin et al. 2020; Beloribi-Djefaflia et al. 2024). *FIG4* mutations are associated with CMT4J, ALS11 (Yilihamu et al. 2022), bilateral temporooccipital polymicrogyria, and Yunis-Varon syndrome (Kitani-Morii and Noto 2020; Deneubourg et al. 2022). Polymicrogyria is characterised by abnormal brain development, resulting in seizures and intellectual disability, whereas Yunis-Varon syndrome is a rare autosomal recessive disorder with skeletal and neurological abnormalities.

*FIG4* mutations, particularly autosomal dominant ones, are associated with ALS11, a severe form of ALS characterised by a combination of progressive motor neuron degeneration and peripheral neuropathy. These mutations often overlap symptomatically with peripheral neuropathies, such as CMT. Abnormal FIG4 causes defects in RNA metabolism, axonal transport, and protein aggregation, which all contribute to motor neuron degeneration (Martin et al. 2020; Beloribi-Djefaflia et al. 2024). *FIG4* mutations were found in 3% of a European ALS cohort, including both familial and sporadic cases (Osmanovic et al. 2017). *FIG4*-associated ALS shows diverse phenotypes, implying the presence of genetic and environmental modifiers (Beloribi-Djefaflia et al. 2024).

The autosomal recessive CMT4J is caused by mutations in *FIG4*, leading to rapidly progressive sensory and motor deficits due to disruption of endosomal-lysosomal trafficking (CMT Association 2025). Mutations, such as *c.122 T > C (p.Ile41Thr)*, impair FIG4 stability and function, resulting in diverse phenotypes from severe early-onset forms to milder late-onset presentations. Clinical findings include demyelination, characterised by reduced NCV, and axonal degeneration, as shown by decreased motor action potentials in electrophysiological studies (Nam and Choi 2019; Beloribi-Djefaflia et al. 2024).

ALS and CMT caused by *FIG4* mutations share common features. Firstly, axonal transport disruption leads to impaired trafficking of organelles and vesicles, compromising neuronal communication and survival (Osmanovic et al. 2017). Secondly, lysosomal dysfunction leads to defective clearance of cellular debris, which exacerbates neurodegeneration (Martin et al. 2020). Thirdly, cytoskeletal instability due to protein aggregation further aggravates neuronal conditions (Beloribi-Djefaflia et al. 2024).

#### Mitochondrial dysfunction

Mitochondria are essential for cellular energy metabolism, and their dysfunction is a common mechanism of neurodegeneration (Johri and Beal 2012). Accordingly, *MFN2* and *CHCHD10* mutations have been implicated in both ALS and CMT (Ait-El-Mkadem Saadi et al. 1993; Marchesi et al. 2011), causing aberrant mitochondrial morphology. *MFN2* mutations disrupt mitochondrial fusion and distribution, leading to energy deficits and contributing to axonal degeneration in CMT (Kitani-Morii and Noto 2020) and motor neuron loss in ALS (Baloh et al. 2007; Abati et al. 2024). Mutations in *CHCHD10* disrupt the cristae structure, impairing oxidative phosphorylation and ATP production. Additionally, mutant CHCHD10 aggregates, impairing mitochondrial dynamics and cellular stress response (Bannwarth et al. 2014; Auranen et al. 2015).

#### MFN2

*MFN2* encodes mitofusin 2, a protein essential for mitochondrial fusion, transport, mitophagy, and interactions with the endoplasmic reticulum (ER). These processes are critical for mitochondrial shape, distribution, and function, which differ based on cell type, physiological conditions, and pathological states (Marchesi et al. 2011; Vinciguerra et al. 2023). Mitofusins mediate mitochondrial fusion and maintain the balance between fusion and fission, thereby influencing mitochondrial morphology and function. Mitochondrial abnormalities associated with *MFN2* mutations include disrupted cristae, impaired membrane potential, and altered oxidative phosphorylation. These pathomechanisms underline the axonal degeneration observed in both CMT2A and ALS. *MFN2* mutations have been linked to CMT2A, HMSN VI, optic atrophy, and ALS (Marchesi et al. 2011; Abati et al. 2024). MFN2 interacts with TDP-43, implicated in mitochondrial dysfunction in ALS, suggesting overlapping pathophysiological pathways.

A case report highlighted the genetic and pathophysiological overlap between ALS and CMT caused by the *c.581 A > C (p.Asp194Ala)* mutation in *MFN2* (Vinciguerra et al. 2023; Abati et al. 2024). This case demonstrates how the same genetic mutation can manifest as distinct neurological disorders, such as ALS-FTD and CMT2A, emphasising the central role of mitochondrial dysfunction in these diseases (Vinciguerra et al. 2023). Individuals with *p.Asp194Ala* show progressive motor weakness and cognitive impairment in ALS-FTD phenotypes, along with axonal degeneration typical of CMT2A. In one family, the mother presented with severe ALS-FTD symptoms, including bulbar involvement, while her son exhibited sensory-motor polyneuropathy consistent with CMT2A (Vinciguerra et al. 2023). An Italian cohort study revealed that 2.3% of patients with ALS carried rare *MFN2* mutations classified as pathogenic or likely pathogenic. These mutations are associated with diverse phenotypes, including classic ALS and ALS-FTD (Abati et al. 2024).

*MFN2* mutations are predominantly associated with CMT2A (Kitani-Morii and Noto 2020), supported by extensive clinical evidence documented in databases, such as OMIM and ClinVar (Baloh et al. 2007). Patients typically present with distal motor and sensory neuropathy; some cases also exhibit optic atrophy and pyramidal tract signs. For instance, *c.839G > A (p.Arg280His)* has been implicated in both CMT2A and ALS, further highlighting the pleiotropic effects of *MFN2* mutations (Marchesi et al. 2011).

*MFN2* mutations exhibit pleiotropy, causing ALS, CMT, or overlapping phenotypes such as ALS-FTD. Both ALS and CMT share mitochondrial dysfunction as a cellular mechanism in some of their subtypes. Specific mutations, such as *p.Asp194Ala* and *p.Arg280His*, disrupt mitochondrial dynamics, leading to axonal degeneration in CMT2A and motor neuron loss in ALS patients. Thus, the same mutation can manifest differently, depending on both genetic and environmental factors.

#### CHCHD10

The *CHCHD10* (coiled-coil-helix-coiled-coil-helix domain-containing 10) gene encodes a mitochondrial protein essential for maintaining cristae and mitochondrial function. It is localised in the intermembrane space and interacts with components of the mitochondrial contact site and cristae-organising system (MICOS) (Bannwarth et al. 2014; Auranen et al. 2015; Brockmann et al. 2018). Since CHCHD10 is crucial for oxidative phosphorylation and mitochondrial DNA integrity, *CHCHD10* mutations disrupt energy metabolism and cellular homeostasis (Ait-El-Mkadem Saadi et al. 1993). CHCHD10 interacts with other mitochondrial proteins, such as mitofilin and CHCHD3, which are involved in mitochondrial genome stability and electron transport chain assembly (Bannwarth et al. 2014). *CHCHD10* mutations cause mitochondrial fragmentation, decreased oxidative phosphorylation, and instability of the mitochondrial genome. *CHCHD10* dysfunction overlaps with other genes, such as *MFN2*, which is involved in mitochondrial dynamics (Abati et al. 2024). Neurological diseases associated with mutations in *CHCHD10* include ALS, FTD, CMT2, mitochondrial myopathy, and spinal muscular atrophy, Jokela type (SMAJ) (Ait-El-Mkadem Saadi et al. 1993; Brockmann et al. 2018; Harjuhaahto et al. 2025).

Mutations in *CHCHD10* have been linked to fALS and FTD, with the *p.Ser59Leu* variant being associated with ALS-FTD phenotypes. This mutation disrupts mitochondrial dynamics, leading to cristae disorganisation (Bannwarth et al. 2014; Auranen et al. 2015). Pathological features include mitochondrial dysfunction, protein misfolding, and the accumulation of insoluble aggregates in motor neurons (Ait-El-Mkadem Saadi et al. 1993). Other variants, such as *c.176 C > T (p.Arg15Leu)* and *c.225 C > T* (*p.Ala72Val)*, have occasionally been identified in patients with ALS. However, evidence supporting their pathology is less robust, as they often display variable penetrance or are sometimes found in cases with atypical ALS presentation (Bannwarth et al. 2014; Consortium 2018). For example, although *p.Arg15Leu* has been described in fALS with slow disease progression and lower motor neuron involvement, its presence in unaffected carriers suggests a role for additional genetic or environmental modifiers (Consortium 2018). The *p.Ala72Val* mutation has been identified in a small subset of ALS patients (Consortium 2018).

A case study found a mutation in *CHCHD10*, *c.197G.T (p.Gly66Val)*, which is strongly associated with CMT2. This mutation, separately characterised in ALS (Brockmann et al. 2018), is particularly common in Finland and sometimes associated with ALS-like phenotypes. Affected individuals present with progressive distal muscle weakness, sensory abnormalities, and mitochondrial dysfunction. Symptoms typically appear in mid-to-late adulthood and progress gradually. Muscle MRI and biopsy showed aberrant mitochondrial organisation and abundant mitochondrial DNA deletions (Auranen et al. 2015).

Patients with *CHCHD10* mutations may thus present with overlapping clinical features of ALS and CMT, such as muscle weakness, atrophy, and sensory deficits; indicating some pathological overlap (Ait-El-Mkadem Saadi et al. 1993; Abati et al. 2024). For instance, both diseases feature mitochondrial dysfunction as a central pathology, driven by disrupted cristae integrity and ATP production (Brockmann et al. 2018). Defective mitochondria impair axonal transport, contributing to the degeneration of both motor and sensory neurons.

#### Epigenetics and the environment in ALS and CMT

While genetic mutations provide a foundation, epigenetic factors additionally influence gene expression and disease progression, adding complexity to disease aetiology. Epigenetic mechanisms regulate gene expression without altering the DNA sequence. Aberrant DNA methylation, histone modifications, and non-coding RNA dysregulation have been implicated in both ALS and CMT. For example, *C9orf72* hypermethylation is a well-documented epigenetic marker of ALS (Xi et al. 2013), whereas histone deacetylase 6 (HDAC6) inhibition is a promising treatment target in CMT (Guo et al. 2017); while the mechanism of HDAC6 *per se* is more cytosolic than epigenetic. These findings highlight the importance of epigenetic regulation in neurodegeneration and its potential for therapeutic intervention (Yamaguchi et al. 2021b).

Both ALS and CMT exhibit phenotypic heterogeneity, suggesting the presence of modifiers that determine disease characteristics beyond the wide spectrum of genetic mutations. A review by *Yamaguchi et al. (2021)* summarised recent studies linking ALS and CMT to epigenetic regulation, with an emphasis on approaches using *Drosophila* models. A limitation of *Drosophila* models is the evolutionary distance of fruit flies from humans; however, they are a good genetic model for understanding neurodegenerative diseases (Kitani-Morii and Noto 2020; Yamaguchi et al. 2021b).

Aberrant DNA methylation has been identified in patients with ALS. One of the best-studied epigenetic modifications in ALS is the hypermethylation of CpG islands near the G4C2 repeat expansion in the *C9orf72* gene, which is also associated with FTD (Xi et al. 2013; Zhang et al. 2021). Another example is the upregulation of DNA methyltransferases Dnmt1 and Dnmt3a in the spinal cords of patients with ALS. Experimental evidence has shown that inhibiting DNA methylation can protect motor neurons from degeneration (Yamaguchi et al. 2021b).

Histone modifications, crucial for chromatin structure and transcriptional regulation, have been reported in patients with ALS. Histone acyltransferases (HAT), such as CBP, p300, and ELP3, have been implicated in ALS pathology. HDACs, particularly HDAC6 (a major deacetylating enzyme of α-tubulin), have been linked to protein aggregation and axonal transport defects, and HDAC inhibitors (HDACi) are being explored as potential therapeutic agents. Furthermore, abnormalities in histone methylation, such as dysregulated H3K9 and H3K27 methylation, have been observed in ALS patients carrying *C9orf72* expansions (Bennett et al. 2019; Yamaguchi et al. 2021b; Di Martino et al. 2024).

Non-coding RNAs, including microRNAs (miRNAs) and long non-coding RNAs (lncRNAs), are important regulators of gene expression in ALS. For example, the miRNAs miR-155 and miR-142 target *UBQLN2*, an ALS-related gene, whereas miR-206 is upregulated in ALS model mice, targeting *SOD1* (Figueroa-Romero et al. 2016; Di Martino et al. 2024). Additionally, lncRNAs such as NEAT1 and MALAT1 can be misregulated in ALS and FTD (Yamaguchi et al. 2021b).

Research linking epigenetic modifications to CMT remains more limited. HDAC6 has been implicated in multiple CMT subtypes, and HDAC6 inhibition reversed axonal loss in a mouse model of mutant *HSPB1*-induced CMT, making it a potential therapeutic target (Guo et al. 2017). Non-coding RNAs have been identified as potential regulators in CMT. miR-149 was identified as a genetic modifier of CMT1A, affecting several genes, including *PMP22* (Nam et al. 2018b). Furthermore, lncRNAs, such as CR18854 and CR43467, interact with *FIG4*, a gene that plays a role in both ALS and CMT (Yamaguchi et al. 2021b). Collectively, these pieces of evidence suggest a possible shared regulatory pathway involving lncRNAs in both diseases.

In addition to genetic and epigenetic factors, environmental factors contribute to the progression of ALS and CMT. Physical activity can modulate the progression of axonal damage, which can delay motor impairment, whereas exposure to harmful chemical agents can increase neuronal stress, accelerating disease progression. Nutritional deficiencies, such as low levels of vitamins or antioxidants, increase oxidative stress, promoting axonal degeneration (Yu et al. 2014; López-Pingarrón et al. 2023). Investigating how epigenetic and environmental modifiers affect protein interactions, vesicle trafficking, and neuronal stress pathways could further reveal potential common mechanisms for both ALS and CMT. Understanding these modifiers could allow for better prediction of disease outcomes.

### Implications and future directions

The genetic commonalities between rare subtypes of fALS and CMT suggests partially shared neurodegenerative pathways, despite the fact that ALS primarily affects the CNS and CMT affects the PNS (Martin et al. 2020; Nemeth et al. 2024). In addition to the possible mechanisms described above, disruption of neuromuscular junctions may be involved across neurodegenerative diseases affecting the motor system (Nemeth et al. 2024). Increasing evidence indicates that many neurodegenerative diseases share overlapping mechanisms, with an aging population leading to increased healthcare costs (Rayamajhi et al. 2024). Understanding shared mechanisms across neurodegenerative disorders could help refine disease classification models. Clinically, genetic screening across disorders could aid in early diagnosis (Olsen et al. 2022; de Boer et al. 2023). Advances in next-generation and whole-exome sequencing will further refine genetic risk assessment and early detection strategies (Lupski et al. 2010; Pang et al. 2017). Both ALS and CMT patients are often diagnosed late or initially misdiagnosed, as early symptoms are typically mild and insidious (Albuquerque et al. 2023; Gwathmey et al. 2023). Early genetic testing of at-risk individuals, such as those with a family history of ALS or CMT, could promote early intervention and improve prognosis.

In addition to the genes described in detail above, evidence exists for involvement of the *GJB1* and *DHTKJD1* genes in both ALS and CMT (Marchesi et al. 2011; Feng et al. 2018; Osmanovic et al. 2021). It is likely that a larger group of – thus far unidentified – common denominators exist. An interesting aspect is the identification of oligodendrocytic and CNS myelin pathologies in ALS (Jamet et al. 2024). Biochemically, PNS and CNS myelin are similar, and this could provide another link between CMT and ALS for future studies.

Understanding shared molecular pathways in neurodegeneration can eventually enable the development of therapies and repurposing of current drugs. Several promising therapies are currently under development. For instance, HDACi are being tested for both CMT and ALS (Guo et al. 2017). Other examples being studied for ALS or CMT include mitochondrial targeting drugs (Zinovkin and Zamyatnin 2019; Genin et al. 2023; Zacharioudakis and Gavathiotis 2023), autophagy enhancers (Deneubourg et al. 2022), CRISPR-based gene therapy (Zhang 2021), and RNA-targeted therapies (Mejzini et al. 2019; Mendonsa et al. 2021).

Overall, both gain- and loss-of-function effects can be observed across the ALS and CMT target genes, depending on the affected gene. Artificial intelligence-based models (e.g. AlphaFold3), CRISPR gene-editing techniques, and stem cell models can be used for further investigation and validation of molecular mechanisms (Guo et al. 2017; Peggion et al. 2022). Not all individuals with pathogenic mutations develop the disease phenotype, and there is variability in expressivity and penetrance (sometimes within the same family pedigree), indicating the presence of modifiers (Osmanovic et al. 2017). Investigating how genetic (Chow et al. 2009; Pang et al. 2017), epigenetic (Bennett et al. 2019), and environmental (Yu et al. 2014) modifiers affect protein interactions, vesicle trafficking, and neuronal stress pathways will provide insights into the pathogenesis of both ALS and CMT (Yamaguchi et al. 2021b; Zhang et al. 2021). Longitudinal clinical studies could also be conducted to track patients with the rare but documented ALS-CMT overlap.

Identification of more rare, shared target genes between disorders allows to analyse and/or predict molecular properties and understand potential effects of disease-causing mutations at the protein level. For individual protein targets, structure-function studies can provide detailed insights into disease mechanisms at the molecular level. For CMT, a line of missense mutations in *GDAP1* affects protein stability as a major molecular mechanism (Nguyen et al. 2020; Sutinen et al. 2022, 2023). Of the proteins discussed here, for which little experimental structural data exist, MFN2 can be used as an example. The crystal structure of human MFN2, along with structure-function studies on selected CMT2A variants, suggests diverse effects on MFN2 function (Li et al. 2019).

## Conclusions

We aimed to explore the common molecular features between ALS and CMT, rather than giving an extensive detailed coverage of each study or mutation, to provide an overall view of the field and spark new research ideas. Despite the limitations inherent in a literature and database survey, the recurrence of similar possible pathomechanisms and genetic associations between ALS and CMT across independent studies reinforces the relevance of the findings. While further research is warranted, the consistency of the identified genes/mutations and putative related mechanisms suggests that at least some rare forms of fALS and CMT may share molecular overlaps. Identifying common target genes advances our understanding of how common molecular and cellular dysfunctions may contribute to these disorders. Further research into the commonalities between CNS and PNS neurodegenerative disorders at the molecular level is crucial to understand fundamental biological processes in myelinating cells and axons.

## Data Availability

The search protocol for the literature and database review is available online (Aynaashe and Kursula 2025).

## Abbreviations

ALS: Amyotrophic lateral sclerosis
AOA2: Ataxia with oculomotor apraxia type 2
ARJALS: Autosomal recessive juvenile-onset ALS
ARHSP: Autosomal recessive hereditary spastic paraplegia
CAE: Cryptic amyloidogenic elements
CDCBM13: Complex cortical dysplasia with other brain malformations-13
CIDP: Chronic inflammatory demyelinating polyneuropathies
CMT: Charcot-Marie-Tooth disease
CRISPR: Clustered regularly interspaced short palindromic repeats
DDR: DNA damage repair
dHMN: Distal hereditary motor neuropathy
EMG: Electromyography
ER: Endoplasmic reticulum
ERAD: Endoplasmic reticulum-associated degradation
fALS: Familial ALS
FTD: Frontotemporal dementia
FTLD: Frontotemporal lobar degeneration
HAT: Histone acyltransferase
HDAC: Histone deacetylase
HDACi: Histone deacetylase inhibitor
HMSN: Hereditary motor and sensory neuropathy
HSP: Hereditary spastic paraplegia
IBMPFD: Inclusion body myopathy with Paget's disease of bone and frontotemporal dementia
LMN: Lower motor neuron
lncRNA: Long non-coding RNA
MICOS: Mitochondrial contact site and cristae-organizinng system
miRNA: microRNA
MR: Magnetic resonance
MS: Multiple sclerosis
mtDNA: Mitochondrial DNA
NCV: Nerve conduction velocity
NGS: Next-generation sequencing
PEG: Percutaneous endoscopic gastrostomy
PNS: Peripheral nervous system
ROS: Reactive oxygen species
sALS: Sporadic ALS
sHSP: Small heat shock protein
SMA: Spinal muscular atrophy
SMAJ: Spinal muscular atrophy, Jokela type
STAHP: Progressive spastic tetraplegia and axial hypotonia
TDP-43: TAR DNA-binding protein 43
UMN: Upper motor neuron
WES: Whole-exome sequencing
